# *Pediococcus pentosaceus* IM96 Exerts Protective Effects against Enterohemorrhagic *Escherichia coli* O157:H7 Infection In Vivo

**DOI:** 10.3390/foods10122945

**Published:** 2021-11-30

**Authors:** Haixin Li, Xinqiang Xie, Ying Li, Moutong Chen, Liang Xue, Juan Wang, Jumei Zhang, Shi Wu, Qinghua Ye, Shuhong Zhang, Runshi Yang, Hui Zhao, Lei Wu, Tingting Liang, Yu Ding, Qingping Wu

**Affiliations:** 1 School of Biology and Biological Engineering, South China University of Technology, Guangzhou 510006, China; lihaixinscut@163.com; 2 Guangdong Provincial Key Laboratory of Microbial Safety and Health, State Key Laboratory of Applied Microbiology Southern China, Institute of Microbiology, Guangdong Academy of Sciences, Guangzhou 510070, China; woshixinqiang@126.com (X.X.); liying@gdim.cn (Y.L.); cmtoon@hotmail.com (M.C.); xueliang@gdim.cn (L.X.); zhangjm926@126.com (J.Z.); wushiloveyou@126.com (S.W.); yeqinghua2002@163.com (Q.Y.); zhangshh2001@163.com (S.Z.); yangrunshi1@gmail.com (R.Y.); zhaohuichinese@163.com (H.Z.); wuleigdim@163.com (L.W.); gdim_liangtt@outlook.com (T.L.); 3 College of Food Science, South China Agricultural University, Guangzhou 510642, China; wangjuan@scau.edu.cn; 4 Department of Food Science and Technology, Institute of Food Safety and Nutrition, Jinan University, Guangzhou 510632, China

**Keywords:** *Pediococcus pentosaceus* IM96, EHEC O157:H7, probiotics, inflammation, intestinal barrier, intestinal microbiota

## Abstract

Enterohemorrhagic *Escherichia coli* (EHEC) is a notorious and prevalent foodborne pathogen which can cause serious intestinal diseases. The antagonistic activity of probiotics against EHEC is promising, but most of the studies concerning this subject have been carried out in vitro. Specifically, the interaction between *Pediococcus pentosaceus* and EHEC O157:H7 in vivo has not been reported yet. In this study, we investigated the protective effect of *P. pentosaceus* IM96 on EHEC O157:H7-infected female mice in vivo. The results demonstrated that *P. pentosaceus* IM96 reduced the level of pro-inflammatory factors and increased the level of anti-inflammatory factors of EHEC O157:H7-infected mice. Furthermore, *P. pentosaceus* IM96 alleviated intestinal mucosal damage and increased the level of MUC-2, tight junction (TJ) proteins, and short chain fatty acids (SCFAs). The intestinal microbial community structure and the diversity and richness of the microbiota were also changed by *P. pentosaceus* IM96 treatment. In summary, *P. pentosaceus* IM96 exerted protective effects against EHEC O157:H7 via alleviating intestinal inflammation, strengthening the intestinal barrier function, and regulating intestinal microbiota, suggesting that *P. pentosaceus* IM96 might serve as a potential microbial agent to prevent and treat intestinal diseases caused by EHEC O157:H7 infection in the future.

## 1. Introduction

Enterohemorrhagic *Escherichia coli* (EHEC) is a food-borne pathogen that seriously threatens human health, wherein EHEC O157:H7 is considered as the most common protoserotype since it can cause hemorrhagic diarrhea, enteritis, and hemolytic uremic syndrome (HUS) [1]. At present, the use of antibiotics has been identified to be the most common method to treat EHEC O157: H7 infection. However, recent studies have confirmed the emerge of resistant and even super-resistant bacteria due to the abuse of antibiotics, which will pose a serious threat to human health [2,3]. Therefore, a safe, stable, cost-effective, and biological antibacterial agent is urgently needed to be developed to reduce or even replace the use of antibiotics.

Probiotics are defined as, “live microorganisms that, when administered in adequate amounts, confer a health benefit on the host” [4]. Numerous studies have shown that probiotics have an antagonistic effect on pathogenic bacteria. Additionally, it has been demonstrated that probiotics improve immune regulation, strengthen the intestinal barrier, and balance intestinal microbiota [5]. A previous study showed that probiotic *Bacillus* bacteria comprehensively abolished the colonization of the dangerous pathogen *Staphylococcus aureus* by inhibiting *S. aureus* quorum sensing in a rural Thai population [6]. Bioengineered *Lacticaseibacillus casei* prevented foodborne *Listeria monocytogenes* infection through competitive exclusion, maintenance of intestinal epithelial barrier functions, and contact-dependent immunomodulation [7]. Therefore, probiotics and their metabolites, as potential antibacterial agents, have attracted great interest in the treatment of diseases caused by pathogenic bacteria.

*P. pentosaceus* is a facultative anaerobic lactic acid bacterium that can affect carbohydrate degradation. It is a promising strain with valuable potentials [8]. *P. pentosaceus* can not only be used as biological food preservatives and flavor additives, but it also functions in the fields of food engineering, agriculture, and animal husbandry. *P. pentosaceus* is a potentially predominant probiotic strain due to its anti-inflammatory, anti-cancer, anti-oxidant, detoxifying, and lipid-lowering abilities [9]. Moreover, the antimicrobial effects of *P. pentosaceus* are being highlighted. *P. pentosaceus* I13, *P. pentosaceus* ATCC 43200 and *P. pentosaceus* LJR1 exerted antagonistic effects against *Listeria monocytogenes* in vitro [10,11,12]. The cell free supernatant (CFS) of *P. pentosaceus* 4I1, which was isolated from the freshwater fish, restricted the growth of *Escherichia coli* O157:H7 in vitro [13]. However, the interaction between *P. pentosaceus* and EHEC O157:H7 in vivo has not been researched yet. Therefore, this study explored the protective effect of *P. pentosaceus* IM96 on EHEC O157:H7-infected female mice in vivo.

## 2. Materials and Methods

### 2.1. Strains, Cultures and Growth Environment

*P. pentosaceus* IM96 was isolated from turnip rape, a fermented vegetable food, in Manzhouli, Inner Mongolia, China. *P. pentosaceus* IM96 was cultured in MRS medium (Guangdong Huankai Microbial Sci.&Tech. Co., Ltd., Guangzhou, China). at 37 °C in an anaerobic environment. EHEC O157: H7 ATCC 43895 was cultured at 37 °C in LB medium (Guangdong Huankai Microbial Sci.&Tech. Co., Ltd., Guangzhou, China).

### 2.2. Antibacterial Ability Evaluation

The activated *P. pentosaceus* IM96 was inoculated into an MRS medium, and anaerobically cultured at 37 °C for 24 h. The bacterial suspension was centrifuged at 10,000× *g* for 10 min at 4 °C, and then filtered with a sterile 0.22 µm microporous membrane to obtain the CFS. The activated EHEC O157:H7 ATCC 43895 was inoculated into LB medium and cultured at 37 °C, and 200 rpm for 6 h. The oxford cup agar diffusion method was used to evaluate the antibacterial ability of *P. pentosaceus* IM96 [14]. Firstly, 200 µL of EHEC O157:H7 ATCC 43895 was evenly spread on the nutrient agar plate. Then, 200 µL of CFS of *P. pentosaceus* IM96 was added to the Oxford Cup. CFS was fully diffused at 4 °C and then incubated at 37 °C for 24 h. Finally, the diameter of the inhibition zone was measured, recorded, and pooled from three independent experiments.

### 2.3. Animals and Grouping

Seven-week-old SPF female C57BL/6 mice were obtained from Beijing HFK Bioscience CO., LTD. The mice were placed in a controlled environment with a temperature of 23 ± 3 °C, relative humidity of 55 ± 10%, and light/dark cycle of 12/12 h. During the experiment, the mice were free to fetch water and food. In total, forty mice were used to adapt one week before the experiment and then were randomly divided into four groups. The control group was gavaged with 0.1 mL of PBS, while the EHEC, norfloxacin and IM96 groups were orally administered with 1 × 10^9^ CFU EHEC O157: H7 ATCC 43895. After 4 h, the control group and EHEC were administered intragastrically 0.1 mL PBS, while norfloxacin group was administered intragastrically 5 mg/kg body weight norfloxacin and IM96 group were gavaged 1 × 10^8^ CFU *P. pentosaceus* IM96. The experiment was repeated two times. All experiments were approved by the Institutional Animal Care and Ethics Committee of the Institute of Microbiology, Guangdong Academy of sciences (GT-IACUC202009252) and followed the standard guidelines for maintenance.

### 2.4. Intestinal Morphology Observation

After the experiment, the jejunum and ileum of the mice were quickly fixed in 10% neutral formalin fixative. Then, the tissue was dehydrated, rendered transparent, and embedded with paraffin to prepare tissue sections. The sections were deparaffinized and stained with hematoxylin and eosin (H&E) sequentially. The intestinal morphology was observed with a fluorescence microscope (Olympus IX73, Tokyo, Japan).

### 2.5. Elisa

The jejunum tissue of the mice was homogenized and crushed using a freezer grinder, and the supernatant was collected by centrifugation. The protein content of the jejunum was determined with the BCA protein kit according to the manufacturer’s instruction (Sangon Biotech, Shanghai, China). The mouse Elisa Kits (AngleGene, Nanjing, China) were adopted to measure the level of inflammation factors including IL-1β, IL-6 and TNF-α, IL-10 and intestinal barrier function indicators including MUC-2, Occludin, and ZO-1. Furthermore D-lactic acid (D-LA) and diamine oxidase (DAO) in serum were detected by ELISA Kits (Dogesce, Beijing, China).

### 2.6. Periodic Acid–Schiff (PAS) Staining 

The jejunum tissue was quickly fixed and prepared into paraffin sections with a thickness of 5-μm by referring to the method described above. Following deparaffinization, the sections were stained by PAS staining. Images were obtained using a fluorescence microscope (Olympus IX73).

### 2.7. Immunohistochemistry

The preparation method of the jejunum tissue section is the same as described in 2.6. After deparaffinization, immunohistochemical analyses were performed involving antigen retrieval and incubation with anti-MUC-2 antibody (ab272692) at a dilution of 1:2000, anti-Occludin antibody (ab216327) at a dilution of 1:200, and anti-ZO-1 antibody (ab96587) at a dilution of 1:500, respectively. Then, the sections were incubated with horseradish peroxidase (HRP)-conjugated goat anti-rabbit IgG at a ratio of 1:200 (Servicebio, Wuhan, China). Following DAB staining, images were obtained by a fluorescence microscope (Olympus IX73).

### 2.8. Quantification of SCFAs

SCFAs in the colon contents of the mice was quantified by using a gas chromatograph (GC). Briefly, the colon contents (50 mg) were homogenized in 0.001% vitriol and centrifuged at 13,000× *g* for 25 min at 4 °C and then filtered with a sterile 0.22 µm microporous membrane. Then, 1.0 μL supernatant was loaded on Agilent 7890A (Agilent Technologies, Palo Alto, CA, USA) equipped with TG-624SiIMS chromatographic column (30 m × 0.25 mm × 0.25 um). The supernatant was collected, and the experimental analysis were slightly modified according to Chen et al. [15] under the following conditions: 7.5649 psi pressure, 20 mL/min desolvation gas flow, 3 mL/min cone gas flow, 180 °C oven temperature, and 250 °C for the injector and detector temperature. A volatile acid standard mix (Supelco, Bellefonte, PA, USA) were used as an external standard and to obtain the calibration curve.

### 2.9. Intestinal Microbiota Analysis

Extraction of genomic DNA, PCR amplicon sequencing, and data processing were completed by GENEWIZ, Inc. (South Plainfield, NJ, USA). In brief, genomic DNA from cecal contents of the mice was extracted using DNA extraction Kit (Megan, Guangzhou, China) according to the manufacturer’s protocols. DNA concentration was monitored by Qubit 3.0 Fluorometer. The 16S rDNA V3 and V4 regions were amplified using forward prime CCTACGGRRBGCASCAGKVRVGAAT and reverse primer GGACTACNVGGGTWTCTAATCC. DNA libraries were multiplexed and loaded on an Illumina MiSeq instrument following the manufacturer’s instructions (Illumina, San Diego, CA, USA) and paired-end sequencing was performed. The forward and reverse reads were truncated by cutting off the index and primer sequence and joined with at least 20 bp overlap. Quality filtering on joined sequences was performed and sequence which did not fulfill the following criteria were discarded: sequence length > 200 bp, no ambiguous bases, mean quality score ≥ 20. Image analysis and base calling were conducted by the control software embedded in the instrument. After quality filter and purify chimeric sequences, the resulting sequences were clustered into operational taxonomic units (OTUs) according to Greengenes 13.8 databases (sequences similarity was set to 97%). Related data analysis and graphs were performed using Calypso [16] (http://cgenome.net/calypso/ (accessed on 3 May 2021)) and Microeco bioinformatics cloud (https://bioincloud.tech/ (accessed on 10 May 2021)).

### 2.10. Statistical Analysis

Data analysis was conducted using the unpaired Student’s *t*-test or one-way ANOVA with SPSS 26. Dada were shown as the mean ± SD, and *p*-value < 0.05 were considered statistically significant. Representation of the *p*-value was * *p* < 0.05, ** *p* < 0.01, and *** *p* < 0.001.

## 3. Results

### 3.1. Antibacterial Activity of P. pentosaceus IM96

To verify the antibacterial activity of *P. pentosaceus* IM96, we examined the activity by using the oxford cup agar diffusion method. As shown in Figure S1, the results suggested that *P. pentosaceus* IM96 showed obvious antagonistic activity against EHEC O157:H7 (24.67 ± 0.58 mm).

### 3.2. P. pentosaceus IM96 Improved the Survival Rate and Body Weight of EHEC O157:H7-Infected Mice

**Figure 1:**
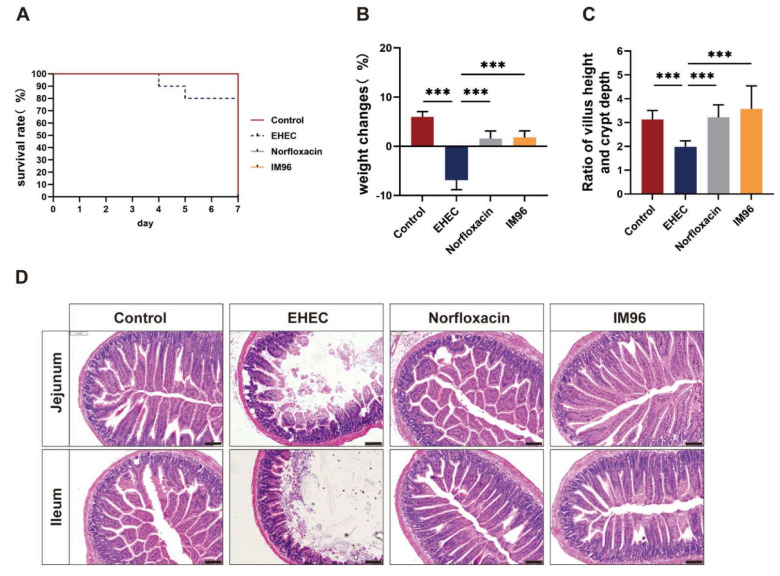
*P. pentosaceus* IM96 relieved the clinical characteristics of EHEC O157:H7-infected mice. (**A**) Survival rate of the mice; (**B**) body weight change of the mice; (**C**) ratio of villus height to crypt depth in jejunum of the mice; (**D**) representative image of H&E staining in jejunum and ileum of the mice (scale bar, 100 μm). All of the data are expressed as the mean ± SD (*n* = 8), *** *p* < 0.001.

During EHEC O157:H7 infection period, the mice of the EHEC group showed lethargy, slowness of motion, and loss of appetite. The survival rate and body weight of the mice were measured and the results showed that the EHEC group had an 80% survival rate. However, there was a 100% survival rate after *P. pentosaceus* IM96 intervention, as with the norfloxacin (Figure 1A). In terms of changes in body weight, EHEC O157:H7 infection significantly reduced the body weight (Figure 1B). Compared with the EHEC group, *P. pentosaceus* IM96 and norfloxacin significantly alleviated the weight loss. There was no significant difference between the IM96 group and the norfloxacin group.

### 3.3. P. pentosaceus IM96 Alleviated Intestinal Damage Caused by EHEC O157:H7 Infection

EHEC infection significantly reduced the ratio of villi height to crypt depth, and *P. pentosaceus* IM96 and norfloxacin intervention significantly increased the ratio of villus height and crypt depth in the jejunum and returned to normal levels (Figure 1C). The intestinal villi of the control group were normal and neatly arranged, and the structure of intestinal mucosa and epithelial cells was complete in the small intestine. In the EHEC group, various intestinal segments had clinical lesions of varying degrees, including villi loss, damage of the intestinal mucosa, and inflammatory cell infiltration, while *P. pentosaceus* IM96 and norfloxacin could improve the intestinal morphology (Figure 1D).

### 3.4. P. pentosaceus IM96 Ameliorated EHEC O157:H7-Induced Intestinal Inflammation

**Figure 2:**
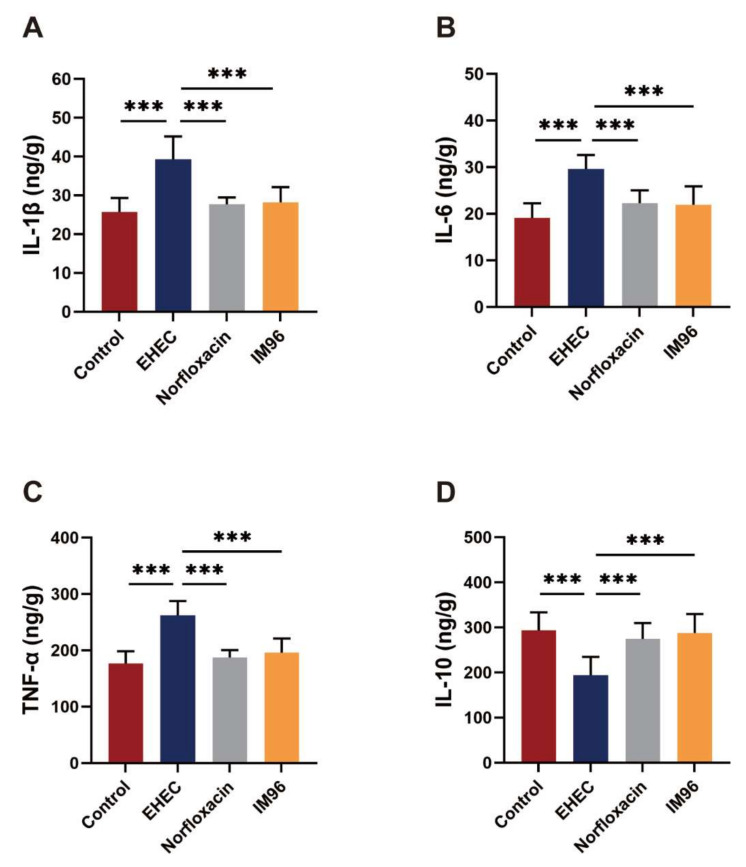
*P. pentosaceus* IM96 relieved EHEC O157:H7-induced inflammation in jejunum of the mice. (**A**) IL-1β; (**B**) IL-6; (**C**) TNF-α; and (**D**) IL-10. All of the data are expressed as the mean ± SD (*n* = 8), *** *p* < 0.001.

The results showed that compared with the control group, the EHEC group had significantly increased the concentrations of pro-inflammatory factors IL-1β, IL-6 and TNF-α (Figure 2A–C). *P. pentosaceus* IM96 treatment could significantly reduce the levels of IL-1β, IL-6, and TNF-α to normal levels with similar effect to norfloxacin. Furthermore, we also measured the concentration of anti-inflammatory factor IL-10 (Figure 2D). After oral administration of EHEC O157:H7, the production of IL-10 was inhibited. Interestingly, after *P. pentosaceus* IM96 and norfloxacin treatment, the secretion of IL-10 was significantly increased. Additionally, as shown in Figure S2, both *P. pentosaceus* IM96 and norfloxacin can significantly decrease the concentration of IL-1β and IL-6 in the serum.

### 3.5. P. pentosaceus IM96 Restored the Intestinal Epithelial Barrier Function

**Figure 3:**
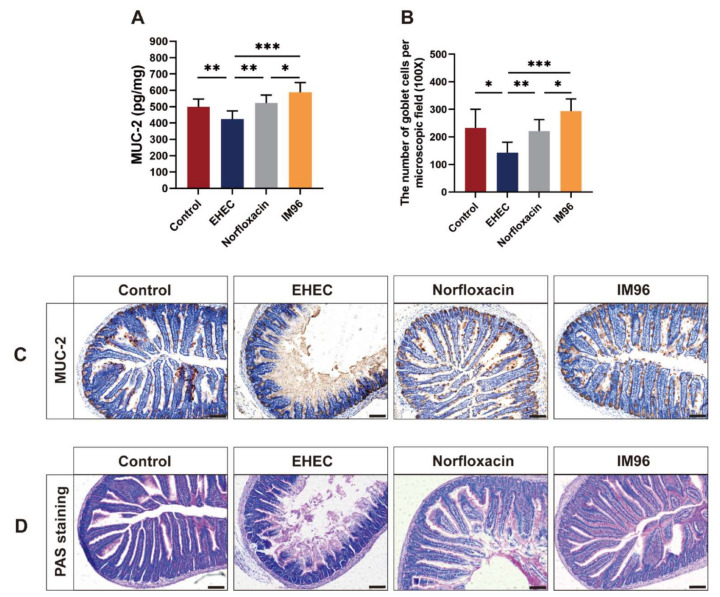
*P. pentosaceus* IM96 enhanced the intestinal epithelial barrier function in jejunum of the mice by upregulating MUC-2 expression. (**A**) The concentration of MUC-2; (**B**) the number of goblet cells per microscopic field (scale bar, 100 μm); (**C**) representative images of immunohistochemical stainings of MUC-2; and (**D**) representative images of PAS stainings (scale bar, 100 μm). All of the data are expressed as the mean ± SD (*n* = 8), * *p* < 0.05, ** *p* < 0.01, *** *p* < 0.001.

EHEC O157: H7 infection could markedly reduce the MUC-2 concentration in the jejunum (Figure 3A). Compared with the EHEC group, both *P. pentosaceus* IM96 and norfloxacin could increase the concentration of MUC-2 in the jejunum. Remarkably, the effect of *P. pentosaceus* IM96 was better than that of norfloxacin on promoting MUC-2 expression in the jejunum. The immunohistochemistry results of MUC-2 confirmed that *P. pentosaceus* IM96 could restore the intestinal epithelial barrier function by upregulating MUC-2 expression (Figure 3C). Furthermore, to further explore the effect of *P. pentosaceus* IM96 on MUC-2, we studied the effect of *P. pentosaceus* IM96 on goblet cells in the jejunum using PAS staining (Figure 3D). The results showed that the number of goblet cells in the IM96 group and the norfloxacin group was notably higher than that in the EHEC group (Figure 3B). Interestingly, *P. pentosaceus* IM96 was superior to norfloxacin regarding the number of goblet cells in the jejunum.

**Figure 4:**
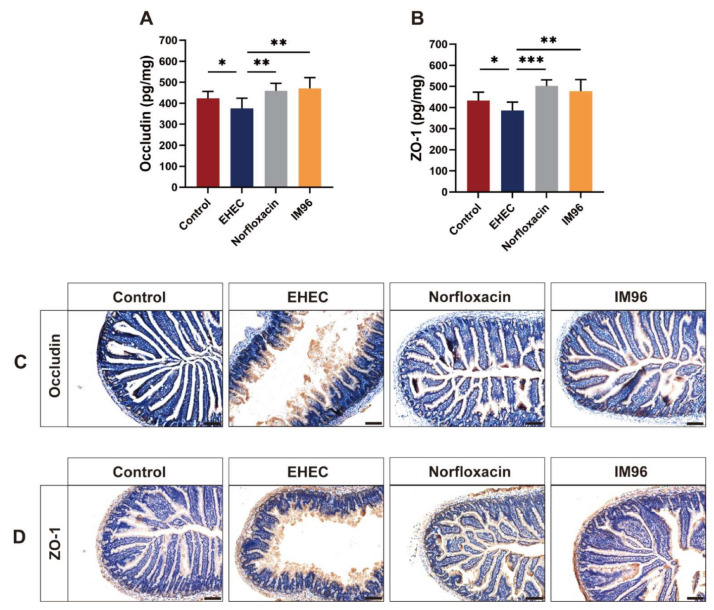
*P. pentosaceus* IM96 enhanced the intestinal epithelial barrier function in jejunum of the mice by increasing TJ proteins expression. (**A**) The concentration of Occludin and (**B**) ZO-1; (**C**) representative images of immunohistochemical stainings of Occludin and (**D**) ZO-1 (scale bar, 100 μm). All of the data are expressed as the mean ± SD (*n* = 8), * *p* < 0.05, ** *p* < 0.01, *** *p* < 0.001.

The concentration of Occludin and ZO-1 was considerably decreased in the jejunum in the EHEC group compared with that in control group (Figure 4A,B). *P. pentosaceus* IM96 and norfloxacin tremendously increased the production of Occludin and ZO-1 in the jejunum compared with that in the EHEC group. The immunohistochemistry results of Occludin and ZO-1 confirmed that *P. pentosaceus* IM96 could restore the intestinal epithelial barrier function by increasing TJ proteins expression such as Occludin and ZO-1 (Figure 4C,D). We also determined the effects of *P. pentosaceus* IM96 on intestinal permeability by detecting the concentration of D-LA and DAO in the serum. As shown in Figure S3, compared with the control group, the EHEC group remarkably increased the concentrations of D-LA and DAO. In contrast, *P. pentosaceus* IM96 effectively improved EHEC O157:H7-disrupted intestinal permeability, as did norfloxacin.

### 3.6. P. pentosaceus IM96 Restored the Concentration of SCFAs in EHEC O157:H7-Infected Mice

**Figure 5:**
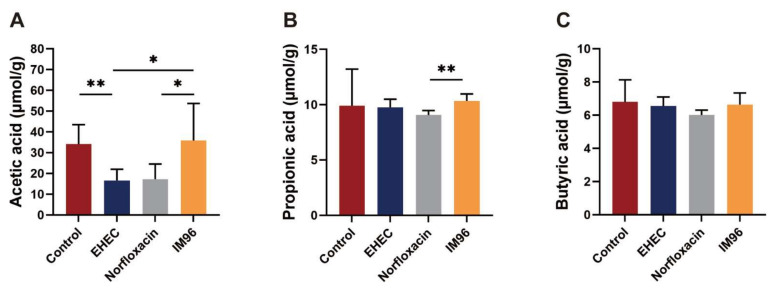
*P. pentosaceus* IM96 return the concentrations of SCFAs to normal levels. (**A**) Acetic acid; (**B**) propionic acid; (**C**) butyric acid. All of the data are expressed as the mean ± SD (*n* = 6), * *p* < 0.05, ** *p* < 0.01.

Compared with the control group, EHEC infection reduced the production of acetic acid. *P. pentosaceus* IM96 increased the concentration of acetic acid to normal levels in the colon of EHEC O157:H7-infected mice (Figure 5A). In addition, the concentration of propionic acid in the IM96 group was also higher than that in the EHEC group (Figure 5B). The concentrations of acetic acid, propionic acid, and butyric acid in the IM96 group were higher than those in the norfloxacin group (Figure 5A–C).

### 3.7. P. pentosaceus IM96 Regulated Intestinal Microbiota Disruption Caused by EHEC O157:H7

**Figure 6:**
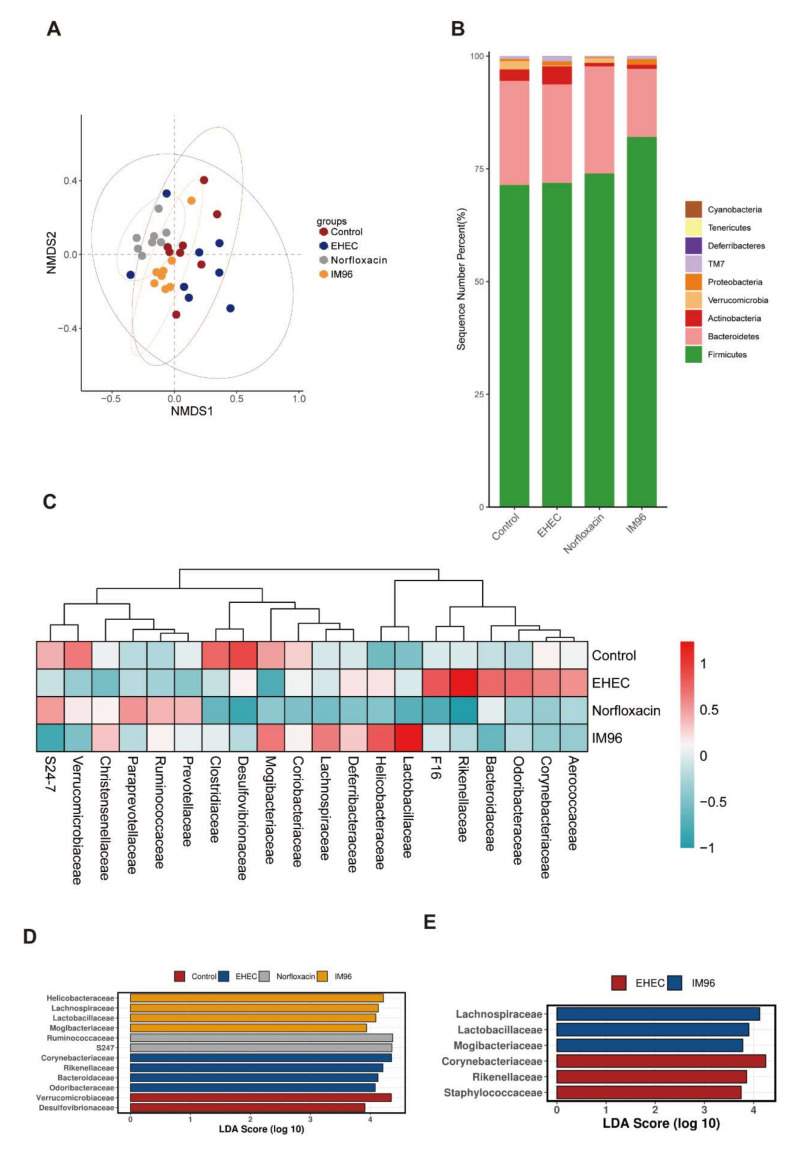
*P. pentosaceus* IM96 regulated the bacterial community of the cecum microbiota. (**A**) NMDS; (**B**) relative abundance richness at phyla level; (**C**) heatmap of the top 20 families; (**D**) LEfSe on family level; and (**E**) LDA in EHEC group and IM96 group.

Chao1, ACE, Richness, and Shannon Index were used to reflect the alpha diversity of the intestinal microbiota. Compared with the EHEC group, *P. pentosaceus* IM96 intervention improved the Chao1, ACE, Richness, and Shannon Index of the intestinal microbiota of the mice, while norfloxacin had the same effect (Figure S4A–D). Nonmetric multidimensional scaling (NMDS) analysis also showed that the four groups were clustered in different places (Figure 6A). The mice in the EHEC group were affected by EHEC infection, which exhibited disordered and scattered distribution of intestinal microbiota. Compared with the EHEC group, the distribution of intestinal microbiota in IM96 group was relatively stable and concentrated, which was close to the control group. The difference of the mice microbiota between the EHEC and IM96 group indicated that IM96 had a positive effect on the intestinal microbiota, making the intestinal microbiota of the mice develop more normally. At the phyla level, Firmicutes and Bacteroides were two most abundant phyla among the four groups (Figure 6B). Compared with the other three groups, the relative abundance of Firmicutes in the IM96 group showed an evident increase, while Bacteroides were reduced. At the family level, Desulfovibrionaceae, Clostridiaceae, Mogibacteriaceae, and Verrucomicrobiaceae were more prevalent in the control group, but Aerococcaceae, Corynebacteriaceae, Odoribacteraceae, Bacteroidaceae, Rikenellaceae, and F16 were more common in the EHEC group. In addition, the abundance of Lachnospiraceae, Mogibacteriaceae, Lactobacillaceae, and Helicobacteraceae were more common in the IM96 group, while Prevotellaceae, Ruminococcaceae, Paraprevotellaceae, and S24-7 were more prevalent in the norfloxacin group (Figure 6C). The linear discriminant analysis effect size (LEfSe) analysis was adopted to explore the abundance of dominant microbe in each group (Figure 6D). In comparison of the EHEC group with IM96 group at the family level, we found the relative abundance of Lachnospiraceae, Lactobacillaceae, and Mogibacteriaceae were statistically higher in the IM96 group, while Corynebacteriaceae, Rikenellaceae, and Staphylococcaceae were dominant microbe in the EHEC group (Figure 6E).

**Figure 7:**
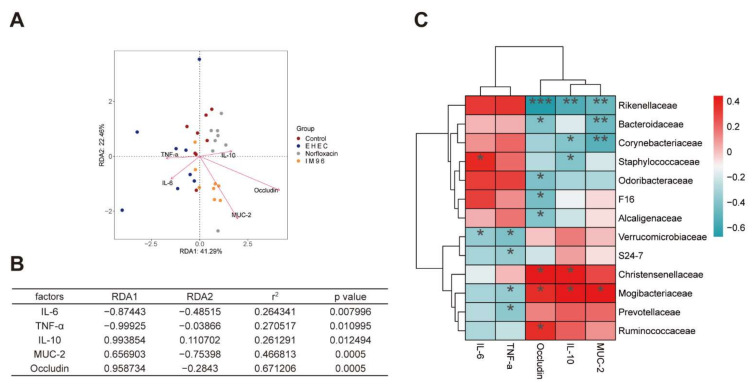
Correlation analysis of environmental factors. (**A**) RDA of intestinal microbiota distribution and environmental factors. The arrows indicate the environmental factors, and the length of the arrow line indicates the degree of correlation between the environmental factor and the sample distribution. The longer the line, the greater the correlation. The angle between the arrow line and the sort axis and the angle between the arrow line indicates the correlation. The smaller the angle, the higher the correlation; (**B**) the significance of environmental factors was analyzed by envfit function. r^2^ is determinant coefficient of environmental factors on intestinal microbiota distribution; (**C**) correlation heatmap, * *p* < 0.05, ** *p* < 0.01, *** *p* < 0.001.

For further investigation of the correlation between the gut microbiota and other sample indicators, the analysis of the environmental factors was adopted to establish a Redundancy Analysis (RDA) model for microbiota samples and five main factors, involving IL-6, TNF-α, Occludin, IL-10, and MUC-2. As shown in Figure 7A, RDA1 and RDA2, respectively, accounted for 41.29% and 22.46% of the whole gut microbiota variation. The coordinate coefficient, correlation, and significance of different factors were listed in Figure 7B, respectively. The result showed that MUC-2 and Occludin were highly correlated with the distribution of gut microbiota. In terms of the correlation between species, Figure 7C showed that Ruminococcaceae, Prevotellaceae, and Mogibacteriaceae were all positively correlated with Occludin, IL-10, and MUC-2, and negatively correlated with IL-6 and TNF-α. Moreover, Rikenellaceae, Bacteroidaceae, Corynebacteriaceae and Staphylococcaceae were all negatively correlated with Occludin, IL-10, and MUC-2, while positively correlated with IL-6 and TNF-α.

## 4. Discussion

Probiotics have been shown to possess the ability to antagonize EHEC O157:H7. EHEC is notorious foodborne pathogen, which is related with many intestinal diseases. To the best of our knowledge, this is the first report on the protective effects of *P. pentosaceus* IM96 against EHEC O157:H7-infected mice in vivo. Our study demonstrated that *P. pentosaceus* IM96 could increase the survival rate and body weight of EHEC O157:H7-infected mice. Consistent with these results, a previous study showed that *Lacticaseibacillus paracasei* NTU 10 could decrease morbidity and increase weight gain in *Escherichia coli* O157:H7-infected mice [17]. Probiotic *Clostridium butyricum* MIYAIRI strain 588 could increase the survival rate when the mice were infected by EHEC O157:H7 [18]. In addition, *P. pentosaceus* IM96 could improve intestinal villi loss, mucosal damage, and inflammatory cell infiltration caused by EHEC O157:H7 in the jejunum of the mice. The ratio of villi height to crypt depth is an important indicator to evaluate the integrity and functional status of the intestinal mucosa. A large ratio indicates that the small intestine has a strong digestive ability. It was reported that *Bacillus amyloliquefaciens* TL106 increased the ratio of villus height to crypt depth in the jejunum [19]. Similarly, we found that compared with the EHEC group, *P. pentosaceus* IM96 significantly increased the ratio of villus height to crypt depth in the jejunum, indicating that *P. pentosaceus* IM96 could repair the mucosa and enhance absorption function in the jejunum.

Probiotics have been confirmed to play a positive regulatory role in the gastrointestinal tract and relieve intestinal inflammation caused by pathogenic bacteria infection [20]. EHEC O157: H7 generally enters the body through oral administration and acts on the intestinal tract to cause infection. The Shiga toxin and LPS secreted by EHEC O157:H7 can cause intense intestinal inflammation, which promotes the expression of pro-inflammatory factors such as IL-1β, IL-6, and TNF-α. In our study, we found that *P. pentosaceus* IM96 could significantly reduce the expression of pro-inflammatory factors including IL-1β, IL-6, and TNF-α, but increase the anti-inflammatory factor IL-10 in the jejunum of EHEC O157:H7-infected mice. Consistent with this finding, a previous study showed that *Lactiplantibacillus plantarum* CCFM1143 treatments could significantly down-regulated TNF-α and IL-6, but up-regulated IL-10 in Enterotoxigenic *Escherichia coli*-infected mice [21].

The intestinal epithelial barrier is essential for normal physiological function. An impaired epithelial barrier can lead to intestinal diseases, such as intestinal pathogen infection, inflammatory bowel disease, obesity, and irritable bowel syndrome [22]. The function of the intestinal barrier largely depends on the mucus layer and TJ proteins [23,24]. The mucus layer is predominantly composed of mucins and intestinal goblet cells. Intestinal goblet cells could secrete mucins that are mainly formed by MUC-2 [25]. Mucins restrict the entry of pathogens by providing a physical-chemical barrier, giving considerable protection to epithelial cells to against pathogens and ensuring integrity of the mucus layer [25,26]. Previous studies suggested that EHEC O157:H7 infection could damage the intact mucus layer and reduce the expression of mucins in the intestine [27,28]. Consistent with these findings, we found that EHEC O157: H7 infection could greatly decrease the concentration of MUC-2 and the number of goblet cells in the jejunum, but the intake of *P. pentosaceus* IM96 could significantly increase the production of MUC-2 and the number of goblet cells in the jejunum of EHEC O157:H7-infected mice. A previous study showed that *Lacticaseibacillus casei* LC2W could increase the expression of MUC-2 at the gene level in EHEC O157: H7-infected mice [29].

TJ proteins, consisted of transmembrane proteins, are located at the top of intestinal epithelial cells [30]. ZO-1 and Occludin are important TJ proteins in intestinal epithelial cells [31]. TJ proteins form a semi-permeable barrier that can limit the admittance of harmful substances, playing an important role in maintaining the permeability of the intestinal epithelial barrier [27]. Orally administered EHEC O157:H7 could decrease the expression of TJ proteins, damage the epithelial barrier, and increase the permeability of the intestine [32]. In our study, we found that *P. pentosaceus* IM96 could relieve the EHEC O157:H7-induced epithelial barrier disruption and increase the concentration of Occludin and ZO-1 in the jejunum. Consistent with this finding, a previous study showed that *Bifidobacterium infantis* could increase the expression of ZO-1 and Occludin in T84 cells [33]. Additionally, *Limosilactobacillus frumenti* could significantly improve the intestinal mucosal integrity by up-regulating tight junction proteins including ZO-1, Occludin, and Claudin-1 in early-weaned piglets [34].

SCFAs are the primary metabolites produced by the anaerobic fermentation of intestinal microbiota, which function as an important part in maintaining human health maintaining human health and intestinal homeostasis [35]. SCFAs can resist the invasion of harmful bacteria by regulating the intestinal microbiota and lowering the pH in the intestine [36]. Also, SCFAs exhibit antioxidant, anti-cancer, and anti-inflammatory activities, and play an important role in maintaining digestion and immune balance [35]. In our research, *P. pentosaceus* IM96 effectively improved the reduction of SCFAs caused by EHEC infection. *P. pentosaceus* IM96 increased the concentration of acetic acid, propionic acid and butyric acid compared with that in the EHEC group and norfloxacin group. These results indicated that *P. pentosaceus* IM96 might exert a protective effect by increasing the content of SCFAs in the intestine.

Probiotics can regulate the intestinal microbiota and maintain the structure of the intestinal community in a balance [37,38]. The results of alpha diversity suggested that *P. pentosaceus* IM96 increased the overall diversity and richness of the cecal microbiota in the mice. NMDS demonstrated that *P. pentosaceus* IM96 could enhance the stability of intestinal microbiota which were closer to the control group than the norfloxacin group. At the phyla level, *P. pentosaceus* IM96 intervention could increase abundance of Firmicutes, but decrease the level of Bacteroides. LEfSe is used to reveal statistically different biomarkers between different groups. According to the linear discriminant analysis (LDA) score histogram at the family level, compared with the EHEC group, the dominant species in the IM96 group was Lachnospiraceae, Lactobacillaceae, and Mogibacteriaceae, while Corynebacteriaceae, Rikenellaceae, and Staphylococcaceae was significantly reduced. As desirable probiotics, Lachnospiraceae can improve the digestive capacity and protect humans from colon cancer, since many species in this family are related to the production of butyric acid [39,40]. Additionally, Lachnospiraceae are negatively correlated with pro-inflammatory factor IL-6 [41]. As we have known, Lactobacillaceae are generally considered as beneficial bacteria that can regulate the intestinal tract and inhibit the proliferation of pathogenic bacteria. Furthermore, Lactobacillaceae are reported to improve the anti-inflammatory ability in high-fat mice [42]. Mogibacteriaceae have been reported to be associated with high-frequency bowel movements and lean body characteristics [43]. In addition, the abundance of Mogibacteriaceae were highly positive with the higher level of HDL, that can reduce BMI and regulate hyperlipidemia [44]. Corynebacteriaeae were reported as harmful bacteria and associated with diarrhea in *Escherichia coli*-induced mice. *Lactobacillus* administration could improve microbial community structure and attenuate *Escherichia coli*-induced diarrhea by decreasing the relative abundance of *Corynebacterium* [45]. Staphylococcaceae are believed to be pathogens or opportunistic pathogens. Probiotic supplementation can reduce the relative abundance of Staphylococcaceae [46]. Hence, *P. pentosaceus* IM96 is capable of selectively elevating the abundance of beneficial bacteria and decreasing the prevalence of pathogens, these results suggested the beneficial effect of *P. pentosaceus* IM96 functions through regulating the intestinal microbiota. In addition, the results of the correlation analysis of environmental factors showed that Mogibacteriaceae were positively correlated with Occludin, IL-10, and MUC-2, while Rikenellaceae, Corynebacteriaceae, and Staphylococcaceae were all positively correlated with IL-6 and TNF-α. These results suggested that Mogibacteriaceae, the dominant species in the IM96 group, could have functions including producing anti-inflammatory effects and enhancing the intestinal epithelial barrier.

In this study, although *P. pentosaceus* IM96 and norfloxacin have similar effects in regulating intestinal inflammation, epithelial barrier function, and intestinal microbiota, we found that norfloxacin could reduce the concentrations of SCFAs such as acetic acid, propionic acid and butyric acid in the colon compared with the IM96 group. Additionally, as antibiotics can cause resistance to intestinal bacteria, gastrointestinal discomfort and destruction of intestinal microbiota, the use of probiotics to prevent and control pathogenic infections has attracted more attention due to its mitigated side effects and safety. The results suggested that *P. pentosaceus* IM96 might be an alternative therapy to the antibiotic in terms of treatment of EHEC O157:H7.

## 5. Conclusions

*P. pentosaceus* IM96 exerts protective effects against EHEC O157:H7 by relieving intestinal inflammation, strengthening the intestinal barrier function, and regulating the intestinal microbiota in infected female mice. Given the in vitro and in vivo effects of *P. pentosaceus* IM96, this bacterium might be used as a potential probiotic and antibacterial agent, and has a promising application prospect in the prevention and treatment of EHEC O157:H7 infection in the future.

## Data Availability

The authors declare that all of the data and the material used in this study are available within this article. All data generated or analyzed in this study can be obtained from the authors upon reasonable request.

## Supplementary Materials

The following are available online at https://www.mdpi.com/article/10.3390/foods10122945/s1. Figure S1: Antibacterial experiment of *P. pentosaceus* IM96 In vitro; Figure S2: *P. pentosaceus* IM96 relieved EHEC O157:H7-induced inflammation in serum of the mice; Figure S3: *P. pentosaceus* IM96 ameliorated the intestinal permeability disrupted by EHEC O157:H7; Figure S4: *P. pentosaceus* IM96 increased the alpha diversity of intestinal microbiota.
