# Supplementary material for: Pediococcus pentosaceus IM96 Exerts Protective Effects against Enterohemorrhagic Escherichia coli O157:H7 Infection In Vivo
Source: Foods. 2021 Nov 30;10(12):2945. doi: 10.3390/foods10122945 (PMC8700651; doi:10.3390/foods10122945)
Supplement: Supplementary file 1 [file foods-10-02945-s001.zip › foods-1456160-supplementary.pdf]

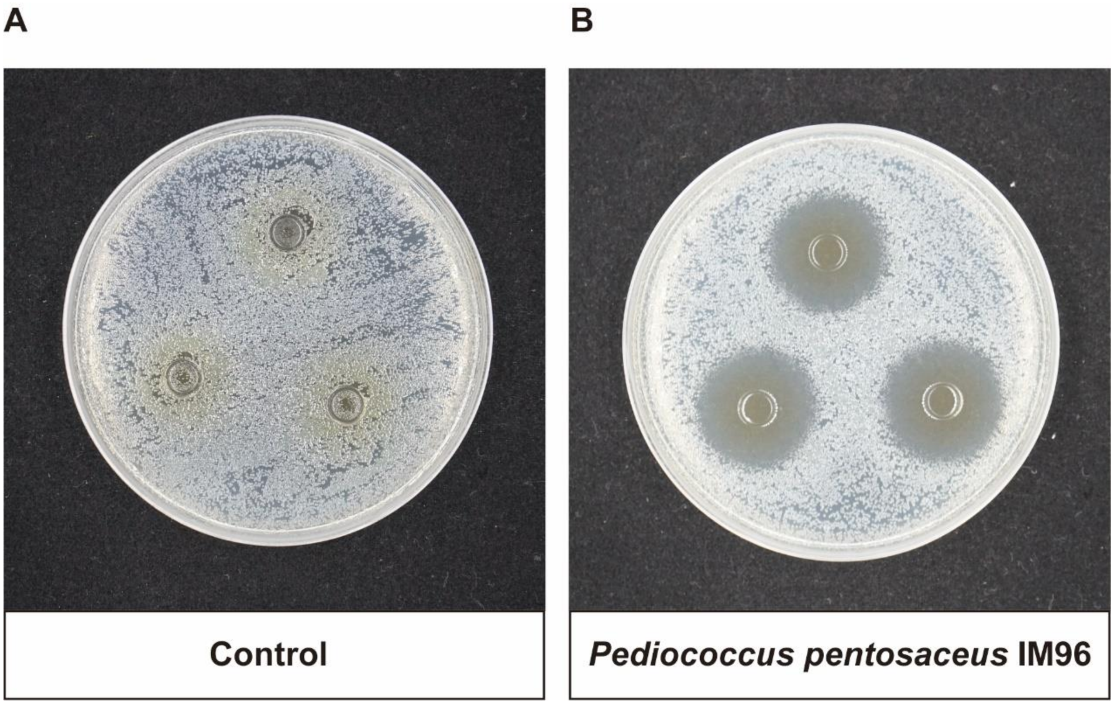

Figure S1. Antibacterial experiment of *P. pentosaceus* IM96 *In vitro*. (A) MRS liquid medium; (B) CFS of *P. pentosaceus* IM96.

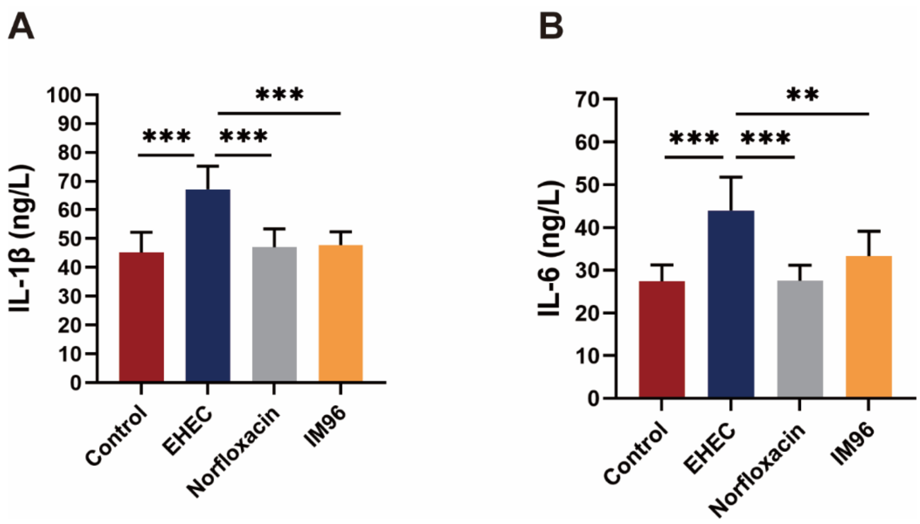

Figure S2. *P. pentosaceus* IM96 relieved EHEC O157:H7-induced inflammation in serum of the mice. (A) IL-1β; (B) IL-6. All of the data are expressed as the mean±SD (n=8), ** $p < 0.01$, and *** $p < 0.001$.

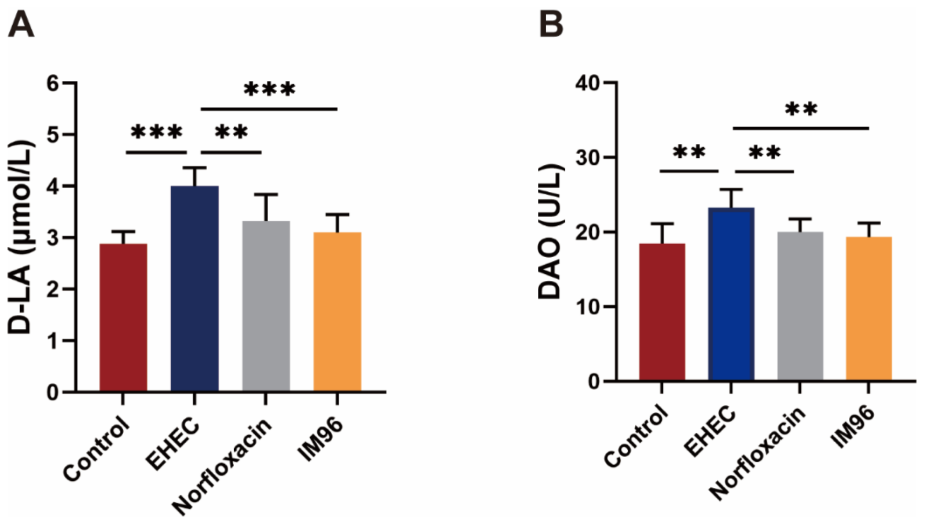

Figure S3. *P. pentosaceus* IM96 ameliorated the intestinal permeability disrupted by EHEC O157:H7. (A) D-LA; (B) DAO. All of the data are expressed as the mean±SD (n=8), ** $p < 0.01$, and *** $p < 0.001$.

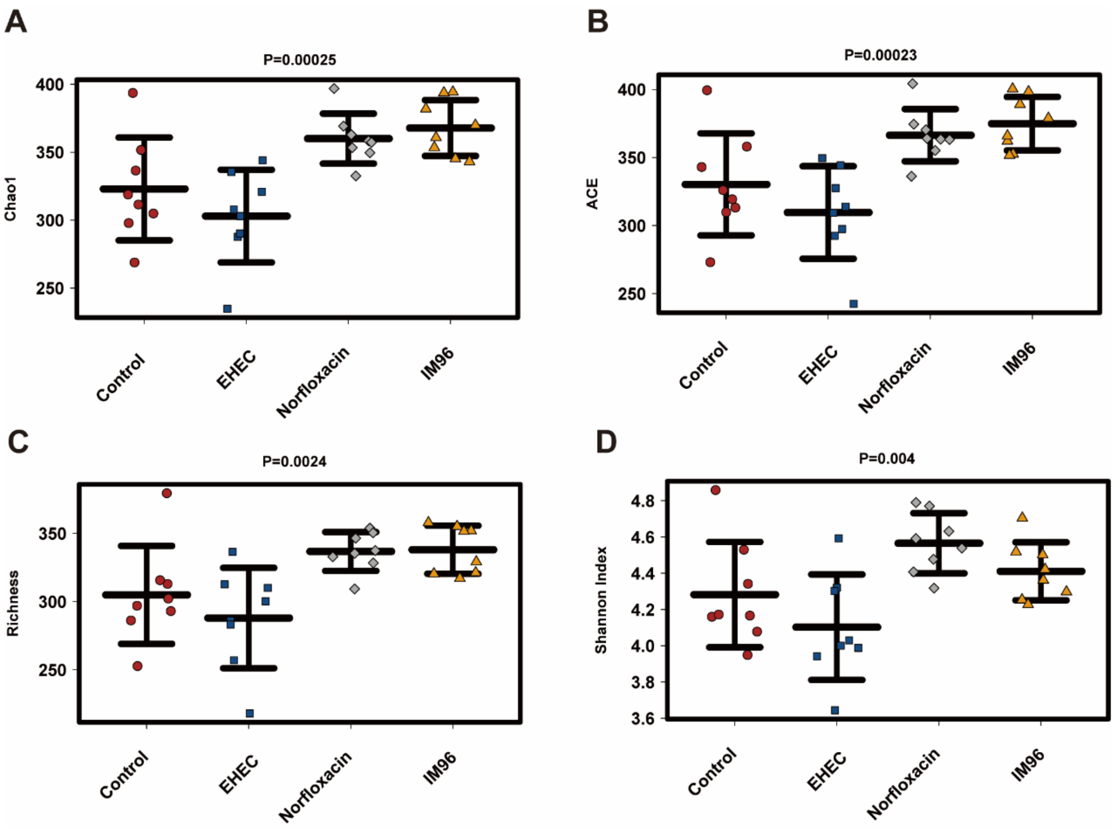

Figure S4. *P. pentosaceus* IM96 increased the alpha diversity of intestinal microbiota. (A) Chao1; (B)ACE; (C)Richness; (D) Shannon index.
